# An assessment of dental caries among young Aboriginal children in New South Wales, Australia: a cross-sectional study

**DOI:** 10.1186/s12889-015-2673-6

**Published:** 2015-12-29

**Authors:** Leanne Smith, Anthony Blinkhorn, Rachael Moir, Ngiare Brown, Fiona Blinkhorn

**Affiliations:** The University of Newcastle, Faculty of Health and Medicine, School of Health Sciences, 10 Brush Road, Ourimbah, NSW 2258 Australia; The University of Sydney, Faculty of Dentistry Westmead Centre for Oral Health, C24A 1 Mons Road, Westmead, NSW 2145 Australia; Department of Indigenous Health and Education, The University of Wollongong, Northfields Avenue, Wollongong, NSW 2522 Australia; National Aboriginal Community Controlled Health Organisation, 3 Garema Pl, Canberra, 2601 ACT Australia

**Keywords:** Aboriginal, Epidemiology, Children, Dental caries, Early childhood caries, Indigenous

## Abstract

**Background:**

Limited research has been undertaken in Australia to assess the dental status of pre-school Aboriginal children. This cross-sectional study records the number of decayed, missing and filled teeth (dmft) and surfaces (dmfs) of pre-school Aboriginal children living in different locations in New South Wales (NSW), Australia.

**Methods:**

A convenience sample of young children from seven Aboriginal communities in rural, remote and metropolitan areas of NSW, was recruited. One calibrated examiner recorded the dmft/s of children with written parental consent.

**Results:**

196 children were invited to participate and 173 children aged two to five years were examined, a response rate of 88.3 %. Forty percent (*n* = 69) of the children were diagnosed with dental caries with a mean of 2.1 (SD = 3.6). The dmft scores were significantly higher in remote locations when compared to rural (*p* = <0.0001) and metropolitan areas (*p* = 0.0155). Children 4–5 years old living in remote NSW had a mean dmft of 3.5 and mean dmfs of 8.0 compared with children living in rural areas who had a dmft and dmfs of 1.5 and 4.2 respectively. Untreated dental caries was the primary contributor to the scores, and children who had previously received dental treatment still had active carious lesions.

**Conclusion:**

There was a high prevalence of untreated dental caries among the Aboriginal children, particularly for those in remote locations.

**Electronic supplementary material:**

The online version of this article (doi:10.1186/s12889-015-2673-6) contains supplementary material, which is available to authorized users.

## Background

Early Childhood Caries (ECC) is a rapid form of dental decay. It affects children under six years of age and typically damages the upper anterior teeth first. It is defined as one or more teeth that are carious, filled or missing due to caries in the primary dentition [1]. It is considered to be severe if it is present on the smooth tooth surfaces of children under three years old [1, 2]. ECC was previously known as ‘baby bottle caries’ as sleeping with a bottle of milk is a major contributor to its aetiology [3]. However ECC is a more inclusive term as there are other factors linked with the disease including frequent consumption of sugary foods/drinks and infrequent tooth brushing with a fluoride toothpaste [4, 5]. Another aspect of ECC is the negative impact it has upon a child’s quality of life [1], causing pain, infection, delayed development, difficulty with eating, multiple psychological disorders [2] and increases the likelihood of caries in the permanent dentition [6–8]. Due to the severity of the disease and the difficulty in providing dental care to young children, many require clinical treatment in a hospital under a paediatric general anaesthetic which does impose risks to the child [9]. Research has shown that Aboriginal children younger than five years of age are hospitalised for dental treatment nearly twice as often as non-Indigenous children [10]. The extent of the caries means that treatment often involves extracting teeth, rather than restoring them. Indeed Aboriginal children have more than twice the number of teeth extracted when compared with other children, with the rate of extractions increasing with remoteness [10].

A recent report in Western Australia found that Australian Aboriginal children between two to four years of age had more extensive dental decay, pain and severe ECC when compared to their non-Indigenous counterparts, with only 30 % of Indigenous children being caries free when compared to 75 % of non-Indigenous children [11].

Information on the prevalence of dental caries among Aboriginal children is lacking in New South Wales. The Child Dental Health Survey [10] undertaken by the Centre for Oral Health Strategy (NSW Health) in 2007 reported that the mean decayed, missing, filled teeth (dmft) scores were substantially higher for five to six year old Aboriginal children (dmft 3.04) when compared to non- Aboriginal children (dmft 1.93) [10]. The main reason for the difference was the level of untreated caries; the mean ‘d’ (decay) for Aboriginal children aged 5–6 years was 2.2 compared with 1.03 for non-Indigenous children. This difference is clearly unacceptable. The NSW Child Dental Health Survey [10] provided valuable information about the dental status of children in New South Wales, however no information was collected on children under five years of age, so data are not available on the prevalence of ECC. Recording the prevalence of ECC in young Aboriginal children is important in order to establish whether preventive strategies are required to control and prevent this oral health problem early in life. Most studies reporting on the prevalence of ECC provide data on children five years of age and older, which is due to the difficulty in screening outside a school environment [9]. The objective of this paper is to report on the prevalence of dental caries of Australian Aboriginal preschool children from seven different rural, remote and metropolitan areas of New South Wales, Australia, in order to provide information on whether targeted oral health promotion programs to control ECC are required.

## Methods

Contact was made with seven Aboriginal Controlled Community Health Services (ACCHS) in rural, remote and metropolitan areas of NSW. A total of 21 preschools including ‘Mums and Bubs groups’ were recommended by the ACCHS for dental screening. Contact was made with the childcare facilities by email and all agreed to partake. A convenience sample of preschool children were invited to participate from the 21 preschools in three remote, two rural and two metropolitan areas of New South Wales, Australia. The children lived in seven Aboriginal communities within an Aboriginal Community Controlled Health Service which had agreed to participate in the research. Within these communities dental examinations were completed in 21 different locations including local preschools, Aboriginal Medical Services (AMS) and childcare facilities including ‘Mums and bubs’ groups from January 2014 to November 2014.

Consent letters outlining the scope of this dental survey were delivered to the participating organisations. Signed consents were collected by a research assistant and only children who were less than six years of age whose parents/guardians had signed the consent letter were eligible to be included in the study. The letter also collected demographic data including Indigenous status.

All dental examinations were undertaken by a highly experienced epidemiologist who was responsible for the examiner training for the NSW State-wide Child Dental Health Survey. Approximately ten percent of the children were re-examined to check for internal examiner consistency. The caries assessments were completed with the child seated, utilising a bright torch with new batteries replaced daily and a mouth mirror. A visual diagnostic system was adopted similar to that used in the NSW 2007 Child Dental Health Survey [10]. The examiner was assisted by an Oral Health Therapist who recorded the caries using the decayed, missing and filled index for teeth (dmft) and individual surfaces (dmfs). The data were collected on paper charts and transferred to a computer based spreadsheet. In addition the Significant Caries Index (SiC) was computed using identical methods to the NSW 2007 Child Dental Health Survey which involves calculating the highest ten percent (SiC^10^) and thirty percent (SiC) of dmft scores [10]. This index was first suggested by Bratthall to represent the caries scores of individuals who have the highest level of dental disease. The SiC Index accounts for the skewed distribution of dental caries in a population and outlines the individuals with substantially higher dmft scores which is not accurately represented by the population mean because of the number of children who do not have any dental health issues [12]. Data analysis was performed using the statistical package R (open source statistical analysis software). The analysis assessed mean, standard deviation (SD), and range of the dmft, dmfs, decayed teeth (d) and missing teeth (m) for the total number of children included in the study and for individual locations (rural, remote and metropolitan). For all health parameters, the Kruskall Wallis test was the most appropriate to test for statistically significant differences between rural, remote and metropolitan populations. When statistically significant results were found, the Wilcoxon Rank-Sum test was used as a post-hoc test to confirm the differences between three sub-groups. Analysis was tested at the 95 % confidence level. The datasets supporting the conclusions of this article are included within the article.

### Ethics approval

Ethical approval was granted by the Research and Development Office, Royal Prince Albert Hospital, Sydney, Protocol Number X14-0101 & HREC-09-RPA 85 (previously X09-0052) and the Aboriginal Health and Medical Research Council Ethics Committee (AH&MRC) Protocol Number 696–09. The research was carried out according to The Code of Ethics of the World Medical Association (Declaration of Helsinki), informed consent was obtained, and the authors institutional review board has approved the study.

## Results

A total of 196 consent forms were issued to Indigenous families and the majority (*n* = 184, 93.9 %) were returned with a positive consent. Eleven (5.6 %) children refused the examination on the day, and 173 were ultimately examined, giving a response rate of 88.3 % [13] (see Additional file 1). A high level of internal examiner consistency was found with a mean kappa score of 0.97. Their ages ranged from two to five years with a mean age of 3.75 years (SD 1.11) with just less than half (79, 45.7 %) being girls.

The mean dmft score was 2.4 (SD 4.1) and the mean dmfs score was 5.3 (SD 10.4). The dmft score ranged from zero to 15, and the dmfs from zero to 44. Untreated dental caries was recorded in forty percent (*n* = 69) of the children.

The Significant Caries Index for the highest ten percent (SiC^10^) and thirty percent (SiC) of children with dental caries was 12.7 and 7.4 respectively. More than forty percent of the children (*n* = 74) were referred for dental treatment. The main reason for referral was dental caries (*n* = 69), followed by fluoride application for incipient lesions (*n* = 4) and trauma (*n* = 1).

For the dmft, a Post Hoc analysis showed a significant difference between rural and remote locations (Wilcoxon Rank Sum Test, W = 1569, *p*-value <0.0001) and metropolitan and remote locations (Wilcoxon Rank Sum Test, W = 715.5, *p*-value = 0.02). However equivalence was found between metropolitan and rural areas (Wilcoxon Rank Sum Test, W = 530.5, *p*-value = 0.34). Table 1 shows that those children living in remote communities had much higher levels of dental caries (d = 3.0; ds = 6.2) when compared to rural (d = 0.6; ds = 1.3) and metropolitan (d = 0.6; ds = 1) areas. The dmft and dmfs scores were higher in remote areas, for example the decay (d) score was 3.0 compared with 0.6 for rural and metropolitan areas. This is a fivefold difference. The SiC^10^ and SiC values were the highest for children aged 4–5 years in remote (SiC^10^ 13.7; SiC 9.9) and rural communities (SiC^10^ 8.0; 4.8). Nine children had a total of 41 teeth extracted due to caries with the majority living in remote areas (*n* = 8; 88.9 %, mean 0.33, SD 1.26). The maxillary incisors were the most common teeth to be extracted. The parameter of missing teeth is equivalent for Indigenous children in all locations (Kruskall Wallis, T = 6.0, *p* = 0.30).

**Table 1:** Mean dmft and dmfs scores for the children examined according to geographical location and mean age

Location	Mean dmft and dmfs scores									
	*n*	Mean (SD) age in years	*d* (SD)	*m* (SD)	*f*	*dmft* (SD)	*ds*	*ms*	*fs*	*dmfs* (SD)
Rural	48	3.3 (1.34)	0.6 (1.7)	0.1 (0.7)	0	0.7 (1.9)	1.3	0.4	0	1.7 (5.4)
Remote	105	4.0 (0.9)	3.0 (4.2)	0.3 (1.3)	0.1	3.4 (4.7)	6.2	1.4	0.1	7.8 (12.2)
Metropolitan	20	3.5 (1.3)	0.6 (1.1)	0 (0)	0	0.6 (1.1)	1.0	0	0	1.0 (2.3)
Total	173	3.8 (1.1)	2.1 (3.6)	0.2 (1.1)	0.1	2.4 (4.1)	4.2	1.0	0.1	5.3 (10.4)

*n* number of children, *d* decayed teeth, *m* missing teeth, *f* filled teeth, *dmft* decayed, missing, filled teeth, *ds* decayed surfaces, *ms* missing surfaces, *fs* filled surfaces, *dmfs* decayed, missing, filled surfaces

Table 2 shows that 4–5 year olds living in remote areas have major problems with untreated caries, the ‘d’ increases with age from 2.9 at 2–3 years to 3.1 at 4–5 years of age (Table 2).

**Table 2:** Mean dmft and dmfs scores for children included in the study according to age and geographical location

Location	Mean dmft and dmfs scores										
	*n*	*d*	*m*	*f*	*dmft*	SiC^10^	SiC	*ds*	*ms*	*fs*	*dmfs*
Rural: 2–3 years	31	0.3	0	0	0.3	3.0	1.2	0.4	0	0	0.4
Rural: 4–5 years	17	1.8	0.3	0	1.5	8.0	4.8	1.2	0.3	0	4.2
Remote: 2–3 years	50	2.9	0.3	0.2	3.4	13.2	9.7	6.2	1.2	1.2	7.5
Remote: 4–5 years	55	3.1	0.4	0	3.5	13.7	9.9	6.3	1.2	0.1	8.0
Metropolitan: 2–3 years	10	0.1	0	0	0.1	1.0	0.3	0.1	0	0	0.1
Metropolitan: 4–5 years	10	1.1	0	0	1.1	4.0	3.7	1.9	0	0	1.9
Total	173	2.2	0.2	0.1	2.3	12.7	7.4	4.2	1.0	0.1	5.3

*n* number of children, *d* decayed teeth, *m* missing teeth, *f* filled teeth, *dmft* decayed, missing, filled teeth, *ds* decayed surfaces, *ms* missing surfaces, *fs* filled surfaces, *dmfs* decayed, missing, filled surfaces

Forty percent (*n* = 69) of the children were diagnosed with dental caries with an average of 2.1 decayed teeth (SD 3.6). There were high caries rates for both the anterior teeth (*n* = 46; 66.7 %) and posterior teeth (*n* = 57; 82.6 %) with 50.7 % (*n* = 35) of children experiencing caries on both anterior and posterior teeth (Table 3). The majority had caries in the maxillary arch (*n* = 62; 89.9 %) with nearly one third having five to ten teeth affected (*n* = 20; 32.3 %). More than half of the children (*n* = 43; 62.3 %) had caries in the mandibular arch with a range of one to seven teeth affected. Many presented with caries in both arches (*n* = 37; 53.6 %).

**Table 3:** The number and proportions of children with carious lesions, together with a breakdown of the affected surfaces

Location				
	*n* (%) with caries	*n* (%) with anterior caries	*n* (%) with posterior caries	*n* (%) with anterior and posterior caries
Rural	8 (11.6 %)	5 (10.9 %)	6 (10.5 %)	3 (8.6 %)
Remote	55 (79.7 %)	40 (87.0 %)	45 (79.0 %)	30 (85.7 %)
Metropolitan	6 (8.7 %)	1 (2.2 %)	6 (10.5 %)	2 (5.7 %)
Total	69 (100 %)	46 (100 %)	37 (100 %)	35 (100 %)

*n* number of children

A small number (*n* = 10) of children had received dental treatment either in the form of restorations or extractions, but all of these children still had active caries on two or more teeth and were referred on for further dental treatment. The majority of these children (*n =* 9; 90 %) had teeth extracted and only two had received any restorative care.

Kendalls non-parametric correlation indicated that the values of dmft, dmfs and decayed teeth have a strong linear relationship. A mild relationship was observed between missing teeth with both dmft and dmfs scores.

## Discussion

There is little published epidemiological data on the caries experience of Aboriginal children, particularly in New South Wales Australia. This study has highlighted three important findings in regards to the dental status of young Aboriginal children. Firstly, nearly half the children (*n* = 69, 40 %) had dental caries, and even the small proportion that had received treatment had new active carious lesions. Secondly, the dmft of the children increased for those living in remote areas with an unacceptable mean dmfs score of up to 8.0 and SiC^10^ of up to 13.7 outlining the disproportionate distribution of decay among those children with the highest level of disease [10]. Finally, there was very little evidence of receipt of dental care and the extraction of compromised teeth was the most common option. The lack of clinical services was highlighted in the 2007 Child Dental Health Survey which also found the prevalence of caries increased with remoteness [8]. Children living in rural and remote areas suffer a heavy burden of disease and chances of receiving dental treatment easily and quickly is limited. Without treatment and prevention, dental caries can lead to the child suffering pain, infection and loss of sleep which also brings unnecessary distress to parents.

A comparative study in Brazil had similar results with just over 50 % of preschool children presenting with ECC. There was a higher prevalence of ECC if the child had poor oral hygiene and reduced socioeconomic development [14]. Children residing in rural areas had higher levels of ECC than those in metropolitan areas. Another study in Queensland, Australia assessed the dmft of preschool children four to six years of age. Similarly, nearly half of the Aboriginal children had dental caries (*n* = 48, 43.7 %) with a mean dmft of 2.44 [15]. ECC has a negative effect on a child and their family. It is associated with reduced quality of lift and impacts on a young child’s physical and psychological health. ECC hinders a child’s ability to eat, talk, sleep and causes dental pain [16]. This study has reported a high prevalence of dental caries (40 %) in young Aboriginal children, which shows that ECC is a serious public health problem with young Aboriginal children suffering a high burden of disease which could be prevented [17]. Despite needing urgent dental treatment, many Aboriginal families in rural or remote areas cannot simply access dental care. This is mainly due to the lack of dental professionals that work outside major cities and the associated travel costs to seek care [18]. Another concern is related to the ‘commonsensical belief’ that if teeth do not hurt then there are no dental problems. Due to this belief and the rapid progression of dental caries in the primary dentition, dental care may not be pursued until a child is in pain and requires extensive dental treatment, which is commonly undertaken in hospital under a general anaesthetic [19–21]. This study showed that more children in rural and remote areas had teeth extracted due to caries, a similar finding to the 2007 Child Dental Health survey results where more teeth were recorded as ‘missing’ than ‘filled’. In this study nine (5.2 %) children had their maxillary incisors extracted early due to caries which compromises their smile and may be associated with psychological trauma, being bullied and low self-esteem [22, 23].

ECC follows a distinct pattern affecting the upper anterior teeth first, and this study found that two thirds (66.7 %) of children with dental decay had active caries on their anterior teeth which is indicative of the frequent intake of sugary drinks. The results from this study show that there is an urgent need for a culturally appropriate oral health promotion program to prevent ECC in young Indigenous children that includes the use of fluoride and contact with a health professional [24, 25]. ECC is influenced by behavioural and social factors. When implementing an oral health promotion program these factors should be modified together as an individual’s behaviour may be influenced by the environment that they live in and changing behaviour alone may not be sustainable [26]. ECC is linked with inappropriate behaviours and can be prevented by brushing twice daily with a fluoride toothpaste and appropriate dietary changes [23, 27]. Previous research shows Fluoride is the cornerstone of caries prevention and is most commonly used in the form of toothpaste and is supported by Cochrane Systematic Reviews [28, 29]. Fluoride toothpaste is a simple and effective way to reduce dental caries in deprived areas [30]. Davies et al. (2002) supplied free toothpaste to families with young children in deprived areas in England. Children were introduced to toothpaste at 12 months of age which resulted in a substantial reduction in dental caries [30]. This was an effective intervention because topical fluoride was introduced at a young age. Appropriate dietary changes include not going to bed with a bottle of milk or other sugary drink, both of which are major contributors to ECC. Reducing the frequency that sugary foods and drinks are consumed is an especially important message to deliver to parents and carers [31].

In this study, most tests showed some degree of difference between remote and rural/metropolitan areas. However the small sample of metropolitan children (*n* = 20) made it difficult to achieve statistically significant results and may be different if the study was repeated with a larger sample. However obtaining permission for surveys of this type is difficult. The results from this study highlight that dental treatment without prevention is of little value in maintaining oral health. For example all of the children in this study who had a previous history of receiving dental treatment had new active carious lesions and had to be referred for further care. Likewise, Fly-in Fly-out dental services that visit remote areas commonly provide emergency treatment and do not focus on prevention [32]. Dental caries can be controlled through simple changes in dietary and brushing behaviours, and these changes are critical for improvement in the long term oral health of young Aboriginal children. Dental caries is a predictive disease, a child with one decayed tooth has a fivefold chance of having other teeth affected when compared with one who is caries free [20]. Caries in the primary teeth also increases the likelihood of children having caries in the permanent dentition. Therefore, preventing the onset of dental caries in the first place is the key to long term good oral health. It is uncommon for a child to visit the dentist before they are two years old [31], however this study shows that some children already have caries by this age and highlights the importance of preventing dental caries from a young age and utilising health care personnel who interact with families with young children to teach preventive messages [19, 31].

There is limited epidemiological data on ECC at a young age because examining young children for caries is a difficult task when compared to recruiting school age children. Researchers tend to focus on older children as gaining access to young children before they start school is difficult, costly and time consuming [9, 33]. In this study 21 locations in seven different geographical areas were visited in order to increase the numbers, as many facilities only cared for a small number of Indigenous children. This study highlights the prevalence of dental caries in young Indigenous children from rural, remote and metropolitan NSW. Nearly half of the children (40 %) had dental caries which indicates current strategies to prevent dental caries are not adequate. This emphasises a need for an oral health promotion program specific for young Indigenous children to prevent dental caries. When implementing an oral health promotion program access to care must be considered. Aboriginal and Torres Strait Islanders who live in rural and remote areas, have poor access to dental care because there is a shortage of dental professionals. A solution to this problem is to train non-dental professionals, for example an Aboriginal Health Worker, to screen for dental caries so an early referral can be organised for a child with oral health problems and provide preventive information to help parents or carers implement appropriate behavioural practices to prevent ECC from starting in the first place [19, 31, 34].

## Conclusion

There was a high prevalence of dental caries (40 %) amongst this sample of young Aboriginal children. All children who had previous dental treatment had untreated carious lesions which required further treatment. Thus, there is a need for an effective oral health promotion program that is easily accessible, culturally appropriate and can be delivered by non-dental personnel to improve the oral health of young Aboriginal children.

## Additional file

Additional file 1:
**Flow diagram explaining recruitment and data available based on STROBE [** 27 **].** Flow diagram explaining recruitment and data available based on STROBE. (DOCX 24 kb)

## Abbreviations

ACCHS: Aboriginal Community Controlled Health Services
AMS: aboriginal medical service
dmfs: decayed, missing, filled surfaces
dmft: decayed, missing, filled teeth
ECC: early childhood caries
SiC: significant caries index
