# Supplementary material for: An assessment of dental caries among young Aboriginal children in New South Wales, Australia: a cross-sectional study
Source: BMC Public Health. 2015 Dec 29;15:1314. doi: 10.1186/s12889-015-2673-6 (PMC4696270; doi:10.1186/s12889-015-2673-6)
Supplement: Additional file 1: — Flow diagram explaining recruitment and data available based on STROBE [ 27 ]. Flow diagram explaining recruitment and data available based on STROBE. (DOCX 24 kb) [file 12889_2015_2673_MOESM1_ESM.docx]

**Figure 1: Flow diagram explaining recruitment and data available based on STROBE [27].**

Excluded (*n =* 23)

- Refused (*n =* 23)

Children attending the sessions and assessed for eligibility

(*n = 196)*

Data available for analysis

- dmft/s (*n = 173)*

Total Recruited

(*n = 173)*
